# A Review of the Effect of Defect Modulation on the Photocatalytic Reduction Performance of Carbon Dioxide

**DOI:** 10.3390/molecules29102308

**Published:** 2024-05-14

**Authors:** Cheng Zuo, Xiao Tang, Haiquan Wang, Qian Su

**Affiliations:** 1 College of Chemistry & Chemical and Environmental Engineering, Weifang University, Weifang 261061, China; 2 College of Science, Nanjing Forestry University, Nanjing 210037, China

**Keywords:** CO_2_ reduction, defect engineering, photocatalysis, charge separation

## Abstract

Constructive defect engineering has emerged as a prominent method for enhancing the performance of photocatalysts. The mechanisms of the influence of defect types, concentrations, and distributions on the efficiency, selectivity, and stability of CO_2_ reduction were revealed for this paper by analyzing the effects of different types of defects (e.g., metallic defects, non-metallic defects, and composite defects) on the performance of photocatalysts. There are three fundamental steps in defect engineering techniques to promote photocatalysis, namely, light absorption, charge transfer and separation, and surface-catalyzed reactions. Defect engineering has demonstrated significant potential in recent studies, particularly in enhancing the light-harvesting, charge separation, and adsorption properties of semiconductor photocatalysts for reducing processes like carbon dioxide reduction. Furthermore, this paper discusses the optimization method used in defect modulation strategy to offer theoretical guidance and an experimental foundation for designing and preparing efficient and stable photocatalysts.

## 1. Introduction

As the economy rapidly expands, a significant amount of fossil fuels is being consumed, resulting in substantial carbon dioxide emissions. These emissions exacerbate the greenhouse effect and disrupt the global ecological balance. Given that fossil fuels are non-renewable energy sources [1], escalating energy consumption and carbon dioxide concentration are having profound impacts on human habitats and the Earth’s ecosystem. Consequently, a pressing global research focus has emerged on how to reduce atmospheric CO_2_ levels and utilize them sustainably. Photocatalysis technology has emerged as a promising solution for converting CO_2_ into crucial chemical fuels such as methane (CH_4_). Although numerous studies have successfully converted CO_2_ into clean fuels, including methanol [2,3,4,5], formic acid [6,7,8,9], and methane [10,11,12,13] via photocatalysis, the yields of these products remain low and are inadequate for industrial-scale applications.

Photocatalysis has undergone a remarkable evolution spanning over a century since its inception. In 1911, the concept of “photocatalysis” was first proposed. Baur et al. [14] validated the ability of ZnO to reduce Ag^+^ to Ag using light energy. TiO_2_’s capacity to fade Prussian blue was also established [15]. A pivotal moment occurred when Honda et al. [16] demonstrated TiO_2_’s capacity for photolytic aquatic H_2_ production, marking a significant milestone in photocatalytic degradation and synthesis reactions. Halman et al. further expanded this field by demonstrating CO_2_ reduction under photocathode catalysis [17]. The researchers went on to achieve photocatalytic CO_2_ reduction with TiO_2_ in water, yielding products like CH_3_OH and CH_4_. Subsequently, metal oxides and sulfides such as SrTiO_3_, WO_3_, ZnO, and CdS were found to exhibit remarkable CO_2_ reduction performance [18,19,20]. In addition, various metal oxides and metal sulfides became mainstream catalysts [21,22,23,24]. Metal–organic frameworks (MOFs) have also emerged as promising photocatalysts, due to their impressive specific surface area and CO_2_ capture capabilities [25]. Recently, Niu et al. [26] introduced a graphite-like g-C_3_N_4_ catalyst that exhibits superior photocatalytic performance.

Ideal photocatalysts for CO_2_ photocatalytic reduction reactions exhibit several key characteristics [27,28,29,30,31,32,33]: (1) a suitable bandgap width, excellent visible and even infrared light absorption ability, and the enhanced utilization of sunlight. (2) Appropriate conduction band and valence band positions to obtain sufficient reduction potential for CO_2_ reduction and O_2_ precipitation potential. (3) A large specific surface area, excellent CO_2_ adsorption performance, and the rapid transport of reactive substances. (4) The fast separation and transport of photogenerated carriers with enhanced carrier separation efficiency. (5) Abundant active sites for CO_2_ reactions. However, single photocatalysts are more or less flawed and it is difficult to achieve the performance of all desirable catalysts. For most catalysts, it is imperative to optimize the performance of the catalyst through certain strategies to compensate for its deficiencies. The activation of stabilized CO_2_ molecules is very difficult, due to the extremely high dissociation energy of the C=O bond (~750 kJ mol^−1^), which inevitably leads to slow reaction kinetics in the photocatalytic reduction of CO_2_ [34]. Defect engineering provides an effective means to improve the performance of photocatalysts. Defect engineering could improve carrier diffusion efficiency, induce electron enrichment, and promote the chemisorption and activation of CO_2_ molecules [35]. Table 1 shows the standard redox potentials for various reactions during CO_2_ reduction [36].

Unlike previous reports in the literature [37,38], this paper offers a comprehensive review of the types of defects introduced in recent photocatalysts, along with their respective mechanisms. Through a detailed analysis of the various components’ roles in the photocatalytic process, this paper elucidates the reasons for the observed improvements in photocatalytic performance. This provides a solid theoretical foundation and experimental guidance for the advancement of novel and efficient photocatalysts. Furthermore, this paper delves into the effects of diverse defect types, such as metallic and non-metallic types, on photocatalyst performance. By combining these theoretical insights with experimental data and reaction principles, it explores their potential applications in the photocatalytic reduction of CO_2_. The mechanisms behind the influence of defect types, concentrations, and distributions on CO_2_ reduction efficiency, selectivity, and stability are revealed. Additionally, this paper surveys the key research hotspots and advancements in this field. The paper also surveys the most prevalent methods to enhance photocatalytic performance, including precise defect concentration control, defect distribution optimization strategies, and the synergistic effects of composite defects. The aim is to provide theoretical guidance and an experimental basis for the design and preparation of highly efficient and stable photocatalysts. Finally, the paper concludes with an overview of the future of defect engineering in photocatalytic CO_2_ reduction.

## 2. Basic Principles of the Photocatalytic Reduction of CO_2_

**Figure 1:**
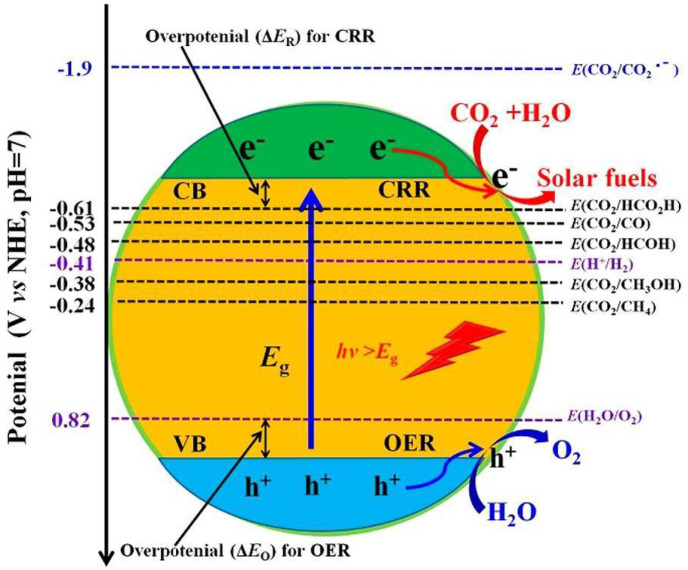
Schematic diagram of the photocatalytic reaction mechanism [39].

The photocatalytic CO_2_ reaction mechanism primarily comprises three stages, exemplified here through conventional semiconductors. As depicted in Figure 1, the initial stage involves the semiconductor catalyst being irradiated by photons exceeding the band gap, triggering electron jumps. They leap to form holes at the top of the valence band and photogenerated electrons at the bottom of the conduction band [39]. Immediately following the second stage is the transfer of the photogenerated electrons and holes; this process must occur swiftly due to the short lifespan of the photogenerated electrons and holes, which is only nanoseconds. To catalyze the reaction, the photogenerated holes and electrons must be promptly transferred to the active surface. In the third stage, the photogenerated electrons on the active surface combine with CO_2_, breaking the C=O bond and reacting with H_2_O to form organic fuel. Concurrently, the photogenerated holes on the active surface react with H_2_O to produce O_2_.

Hence, two prerequisites must be met for the photocatalytic reduction of CO_2_: (i) the photon energy must be equal to or exceed the band gap energy; and (ii) the CB potential must be more negative than the surface electron acceptor potential, while the VB potential must be more positive than the surface electron donor potential. This enables the reaction process of the photocatalytic reduction of CO_2_ to occur [40]. Defect engineering plays an important role in understanding the mechanism of the CO_2_ reduction reaction and improving the performance of CO_2_ photoreduction. Single-component photocatalysts with defects provide excellent prototypes for theoretical calculations. This facilitates an in-depth study of the CO_2_ conversion mechanism and the constitutive relationship. However, some reported that defect engineering strategies for photocatalysts are quite complex and time-consuming. As a result, the conversion efficiency of carbon dioxide is still very low (not just in defective engineered photocatalysts), and there is still a long way to go to achieve industrialization in the future [40].

## 3. Defect Types and the Properties of Photocatalysts

### 3.1. Metallic and Metal-Free Vacancy Defects

ZnS (V_Zn_-ZnS) with metal cation (Zn) vacancies was prepared using an acid-etching strategy by Pang et al. [41] The experimental results show that V_Zn_-ZnS had an excellent selectivity for HCOOH generation in the absence of co-catalysts, averaging above 85%. An examination of the experimental and simulation outcomes reveals that the generation of V_Zn_ led to a reduction in the energy potential barrier, an elevation in surface energy, and a faster charge separation process, ultimately leading to a higher yield of HOOH. A ZnIn_2_S_4_ catalyst was prepared by Jiao et al. [42]. Their findings suggested that the introduction of V_Zn_ was beneficial for elevating the charge density of adjacent S atoms, thus accelerating carrier transfer and separation. This modification significantly shortened the migration time of photogenerated electrons to ~15 ps. The catalyst with V_Zn_ exhibited a higher average CO yield of 33.2 μmol g^−1^ h^−1^, representing a 3.6-fold increase compared to the catalyst without V_Zn_. Huang et al. [43] presented a novel three-phase photocatalytic CO_2_ reduction system by depositing Ag-TiO_2_ nanoparticles at the gas–water interface. This innovation led to a substantial boost in the utilization of visible light and an impressive eight-fold surge in CH_4_ production. Wang et al. [44] alternatively deposited Pt-Cu alloy nanoparticles with varying Pt/Cu ratios onto the TiO_2_ surface. This approach dramatically enhanced visible light absorption through the localized plasma resonance effect of the PtCu alloy, while also elevating the catalytic activity for the photoreduction of CO_2_ to CH_4_. Furthermore, it increased the selectivity of CH_4_ to 100%. Thus, the localized plasma resonance effect of the noble metal could significantly enhance the visible light utilization of the catalyst and obtain ultra-high activity. Fu et al. [45] significantly enhanced CO_2_ adsorption and activation and improved photocatalyst activity after the introduction of Co monoatomic sites in g-C_3_N_4_.

**Figure 2:**
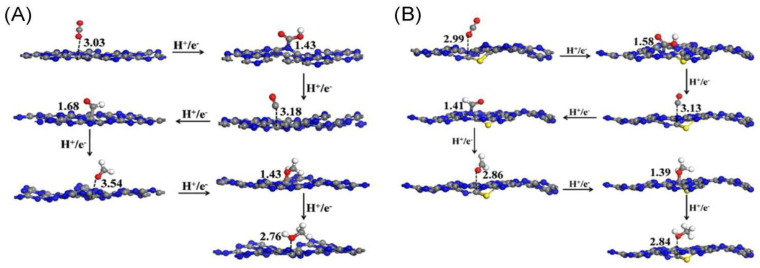
(**A**) Optimal reaction pathways for CO_2_ conversion on g-C_3_N_4_ and (**B**) S-doped g-C_3_N_4_ [51]. Copyright 2015, Elsevier (Amsterdam, The Netherlands).

Carbon nitride (g-C_3_N_4_), a metal-free polymer, has garnered significant attention for its photocatalytic CO_2_ reduction capabilities, due to its simplicity in preparation, remarkable chemical stability, and notably potent reduction potential (E_CB_ ≈ −1.0 eV) [46,47,48,49,50]. However, achieving efficient photocatalytic performance for CO_2_ reduction with g-C_3_N_4_ remains challenging, due to its limited number of active sites. Recent studies have demonstrated that introducing non-metallic defects into g-C_3_N_4_ can generate more active sites, broaden the visible light response, and enhance electron capture, thereby significantly boosting its photocatalytic performance [51,52,53,54]. Figure 2A,B illustrates the optimal reaction pathways for CO_2_ conversion on g-C_3_N_4_ and S-doped g-C_3_N_4_ [51]. Shen et al. [55] successfully prepared g-C_3_N_4_ with carbon vacancies using NH_3_ thermal treatment. This approach enhances catalytic activity for the photocatalytic reduction of CO_2_ by increasing the presence of basic amino groups, elongating the C=O bond lengths, and obtaining stronger CO_2_ adsorption energy.

### 3.2. Metal and Metal-Free Doping Defects

The ideal semiconductor catalyst exhibits a band gap ranging from 1.5 eV to 2.5 eV, while many semiconductors represented by TiO_2_ have band gaps in excess of 2.5 eV. Consequently, the utilization of visible light is limited, necessitating the narrowing of the band gap. This adjustment is commonly achieved through doping techniques, including metallic element doping, non-metallic element doping, and self-doping. These methods introduce impurity levels within the wider band gap, effectively narrowing it and enhancing the utilization of visible and infrared light. Zhang et al. [56] reduced the band gap of TiO_2_ from 3.20 eV to 2.06 eV by doping Cu in TiO_2_. They used the prepared Cu-TiO_2_ for the photoreduction of CO_2_, and the yield of CH_4_ was increased by a factor of 21 in comparison with that of commercial TiO_2_ (P25). Similarly, Xiong et al. [57] prepared N-doped TiO_2_, achieving a reduced band gap of 2.34 eV, significantly improving visible light utilization. Zhang et al. [58] employed a surface hydrogenation strategy to synthesize Ti^3+^ self-doped TiO_2_ mesoporous nanorods, narrowing the band gap to 2.11 eV and enhancing visible light utilization. Collectively, these strategies demonstrate that adjusting the band gap can lead to the improved utilization of visible and infrared light, ultimately resulting in a more efficient photocatalytic reduction of CO_2_.

Abdellah et al. [59] successfully composited the phosphine-phosphated molecular catalyst [Re^I^Br(bpy)(CO)_3_]^0^ with the wide band-gap semiconductor TiO_2_. This integration broadened the light absorption range of TiO_2_ and significantly improved its performance in reducing CO_2_ to CO in visible light. This enhancement was achieved through the rapid electron injection provided by the phosphine-phosphine-phosphated molecular catalyst. To summarize, defect engineering introduces specific defects to the surface and bulk phase of the photocatalyst, such as vacancy, lattice distortion, and crystal surface defects, to regulate its electronic structure and energy level structure, thus significantly affecting the performance of the photocatalyst, including its light absorption capacity, photogenerated carrier separation, and migration efficiency, along with CO_2_ adsorption capacity (Table 2).

## 4. The Effect of Defect Modulation

Defect modulation plays a crucial role in the performance of the photocatalytic reduction of carbon dioxide (CO_2_). Experimental results in recent years have shown that the efficiency, selectivity, and stability of CO_2_ reduction can be significantly improved by precisely controlling the type, concentration, and distribution of defects, as well as their relationship with photocatalytic activity.

### 4.1. Effect of Defect Types on CO_2_ Reduction Efficiency

The choice of defect type has a significant effect on the electronic structure and the formation of active sites of the photocatalyst. Metallic and non-metallic defects can promote CO_2_ reduction by changing the energy band structure and electronic properties of the photocatalysts. Yang et al. [61] showed that oxygen vacancy-modified titanium dioxide, OV-TiO_2_, exhibits little photocurrent under visible light (λ > 420 nm) irradiation. When the oxygen vacancy sites were filled with hydrogen atoms by hydrogenation treatment, it showed greatly enhanced photocurrent under visible light (λ > 420 nm) irradiation, which improved the catalytic activity for the reduction of CO_2_. Graphitic carbon nitride (GCN) can be used as a two-dimensional photocatalyst, and inter-band light absorption can usually be achieved by doping or vacancy engineering [62]. The creation of nitrogen vacancies and oxygen doping in its structure usually leads to a redshift of its absorption edge, while in the case of carbon vacancies, this leads to a blueshift of its absorption edge. However, both the redshift and blueshift in the light absorption of defect-modified GCN showed enhanced photocatalytic performance for CO_2_ reduction [60,63,64].

### 4.2. Effect of Defect Concentration on the Selectivity of CO_2_ Reduction

**Figure 3:**
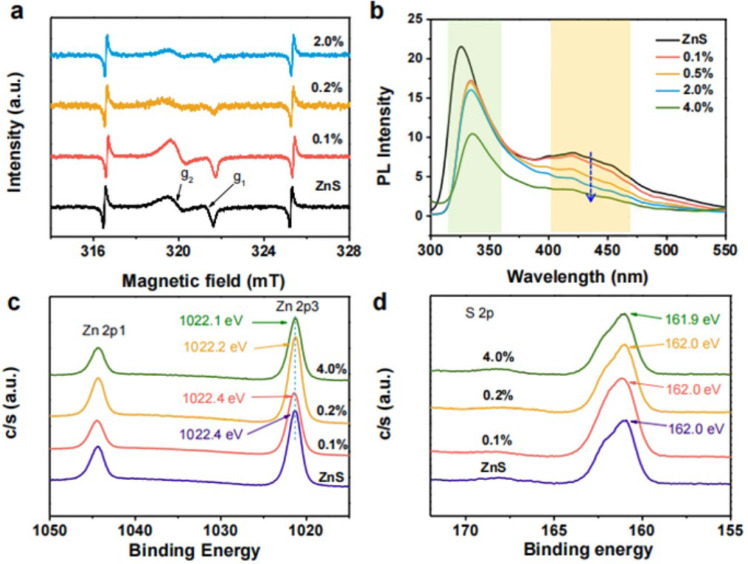
(**a**) ESR spectra and (**b**) PL spectra of undoped ZnS and ZnS:Ni nanocrystals at room temperature. The 425 nm emission that is indicated represents the sulfur vacancy signature. High-resolution XPS spectra of (**c**) Zn 2p and (**d**) S 2p, respectively, of the undoped, ZnS:Ni (0.1%), ZnS:Ni (0.2%), and ZnS:Ni (4.0%) nanocrystals [65]. Copyright 2019, Elsevier.

The precise control of defect concentration is critical to the selectivity of CO_2_ reduction products. Appropriate defect concentrations could provide sufficient active sites while avoiding excessive carrier complexation, thus improving the selectivity of a particular product. Pang et al. [65] reported the production of Ni-doped ZnS (ZnS:Ni) nanocrystals, which were mainly used to enhance the yield of HCOOH. Figure 3a–d shows the changes in the reduction properties of different CO_2_ with different Ni additions. Excessive Ni doping did not guarantee excellent CO_2_ reduction performance. As can be seen in Figure 3b–d, the electron spin resonance (ESR), photoluminescence (PL), and DFT results show that a small amount of Ni-doping produces abundant V_S_, which limits the recombination of photogenerated electrons and holes, while excessive Ni doping can reduce the number of V_S_, which is extremely unfavorable for photocatalytic CO_2_ reduction. Therefore, an appropriate amount of introduced defect concentration is very important for CO_2_ reduction.

### 4.3. Effect of Defect Distribution on the Stability of CO_2_ Reduction

The distribution of defects has an important effect on the stability and long-term activity of photocatalysts. Uniformly distributed defects help to maintain the structural stability of the photocatalyst and reduce the local stress concentration, thus improving the durability of the catalysts. Fu et al. [60] prepared ultra-thin g-C_3_N_4_ nanosheets, which improved the inherent CO_2_ adsorption and activation ability with a larger specific surface area. This significantly enhanced CO_2_ adsorption and activation and improved the photocatalyst activity after the introduction of O-doping in g-C_3_N_4_. In addition, the optimization of defect distribution avoids catalyst deactivation by reducing the aggregation of active sites. 

### 4.4. Relationship between Defect Modulation and Photocatalytic Activity

There is a close relationship between defect modulation and photocatalytic activity. Defects could not only participate in CO_2_ reduction as active sites but also regulate photocatalytic activity by affecting the separation and migration of photogenerated carriers. Shi et al. [66] prepared two-dimensional graphitic carbon nitride nanosheets with nitrogen vacancies (DCN-x, where x denotes the mass of tartaric acid in the preparation process) using the tartaric acid-assisted one-step thermal polymerization of dicyandiamide. The visible light absorption of all the DCN nanosheets containing nitrogen vacancies was enhanced compared with the pristine DCN nanosheets. The change in band gap structure was confirmed by calculating the Kubelka–Munk function. The reduction of the band gap enhances the visible light absorption during photocatalysis. The presence of defects can also promote the effective separation of electron-hole pairs through the formation of intermediate energy bands or the modification of the electronic structure, further improving photocatalytic efficiency. The creation of oxygen vacancies on the WO_3_ atomic layer can introduce intermediate energy band states and extend their photoresponse to visible and even infrared light [67].

The performance of photocatalysts in the CO_2_ reduction reaction can be significantly improved by the rational design and precise control of defect type, concentration, and distribution. These strategies provide an important theoretical basis and experimental guidance for the development of new and efficient photocatalysts, which is of great significance for achieving sustainable energy conversion and environmental purification.

## 5. Optimization Methods for Defect Modulation Strategies

In the research field of the photocatalytic reduction of CO_2_, the optimization of defect modulation strategies is an important way to improve the performance of catalysts. In recent years, researchers have made remarkable progress by designing different types of defects, precisely controlling the defect concentration, optimizing defect distribution, and exploiting the synergistic effect of composite defects.

### 5.1. Optimization Strategies for Defect Distribution

**Figure 4:**
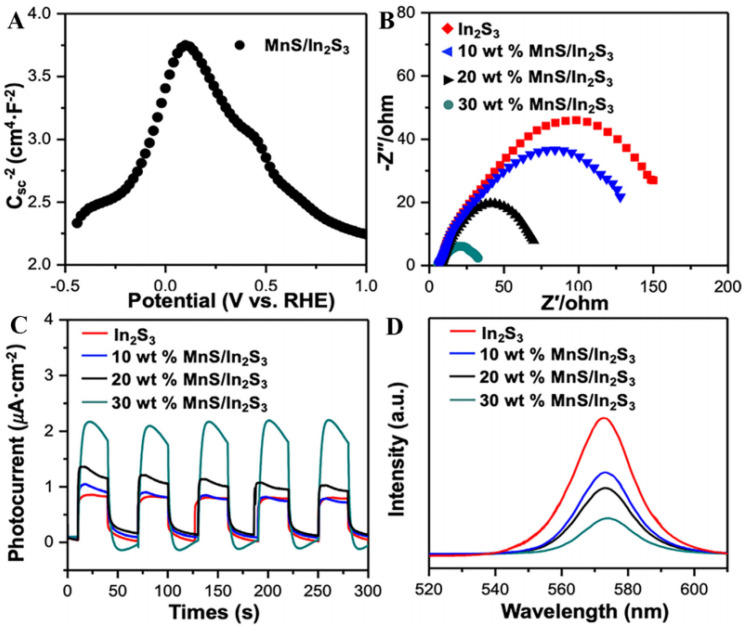
(**A**) M-S plot of the MnS/In_2_S_3_ heterojunction, (**B**) Nyquist plots, (**C**) transient photocurrents, and (**D**) PL spectra of different samples [68]. Copyright 2021, Elsevier.

The distribution of defects is equally important in the performance of photocatalysts. Uniformly distributed defects contribute to the separation efficiency of photogenerated carriers and the stability of the photocatalysts. For example, uniformly distributed defects could be formed on the surface of photocatalysts by specific synthesis methods, such as via solution or template methods. These methods help to improve the activity and selectivity of the photocatalyst while maintaining long-term stability. Tan et al. [68] used the template method to synthesize multilayered MnS/In_2_S_3_ p-n heterojunction nanosheets using Mn^2+^-doped MIL-68(In) as a template and a significant improvement in the activity of the catalysts was obtained, compared to pristine MnS and In_2_S_3_ (Figure 4A,B,D). In addition, the photocatalytic stability of the MnS/In_2_S_3_ heterojunction was tested by performing the reaction four times in succession. After 12 h of testing, no significant decrease in activity was observed (Figure 4C), indicating that the activity of the prepared catalysts was stable. Zhao et al. [69] prepared a Bi@Bi_2_MoO_6_ photocatalyst with excellent catalytic performance, wherein Bi nanoparticles were grown on the surface of Bi_2_MoO_6_ nanosheets with oxygen vacancies. The abundant oxygen vacancies on the surface of Bi_2_MoO_6_ nanosheets not only altered the chemical coordination of the Bi^3+^ ions to form the metal nanoparticles but also facilitated the CO_2_ molecules in their chemisorption on the catalyst surface. The resulting catalyst had strong light absorption efficiency, abundant active sites, and effective spatial separation of photogenerated carriers, and exhibited higher reactivity than the pure Bi_2_MoO_6_ catalyst.

### 5.2. Synergistic Effects of Compound Defects

The introduction of composite defects could realize a synergistic effect between different defects and further enhance the performance of photocatalysts. For example, composite defects can be formed by introducing both metallic and nonmetallic defects in semiconductor materials, and the interactions among these defects can help to improve the separation efficiency of photogenerated carriers and the reduction activity of CO_2_. In addition, the composite defects could also improve the photocatalytic efficiency by modulating the energy band structure of the photocatalysts for better absorption of sunlight. TiO_2_ is one of the most common catalysts in photocatalytic reactions. Liu et al. [70] successfully prepared an Fe-N co-doped TiO_2_ heterojunction photocatalyst (FeTi@C). The results show that the catalyst has a small band gap and excellent visible light absorption. Since the doped N comes from the original MOF, and the radii of Fe^3+^ and Ti^+^ are similar, the Ti-O-Fe tetrahedron and more oxygen vacancies are provided, which improves the photocatalytic efficiency of the CO_2_ reduction of CH_4_. In particular, Fe_0.8_Ti@C has 7.8 and 10.2 times higher CH_4_ yields than the original TiMOF template and the undoped Ti@C, respectively. This synthesis method is a new attempt to harness the synergistic effect of complex defects.

### 5.3. Precise Control of Defect Concentration

The precise control of defect concentration is essential to optimize the performance of the photocatalyst. Defects at too high or low a concentration may lead to the compounding of photogenerated carriers or insufficient active sites. In order to improve the performance of photocatalytic CO_2_ reduction materials, researchers have employed chemical vapor deposition, thermal annealing, and heavy ion irradiation, which are used to control different defect types and concentrations on the catalyst surface. Guo et al. [71] prepared porous ZnO nanosheets (PNS-ZnO) by calcining ZnS nanosheets in air and produced coated g-C_3_N_4_ (g-CN) nanofilms on the surface of PNS-ZnO by thermal deposition. The results show that due to the formation of a heterojunction between PNS-ZnO and g-CN, the defect concentration is uniformly distributed. This leads to significant inhibition of the photogenerated electron and hole complexes, g-CN loading on light absorption, and photocatalytic activity, due to its significant effect on light absorption and photocatalytic activity, prompting all the PNS-ZnO@g-CN nanocomposites to exhibit excellent photocatalytic activity. Among these catalysts, the catalytic activity of PNS-ZnO@g-CN-0.4 appeared to be particularly outstanding. Ding et al. [72] employed single-atom In to modify g-C_3_N_4_ by a single-atom-assisted thermal polymerization process. Single-atom In-bonded N atoms (In^δ+__^N_4_) were constructed on the (002) crystalline surface of g-C_3_N_4_. The results show that the (In^δ+__^N_4_) structure modulates the g-C_3_N_4_ layer spacing and reduces the defect concentration, as well as changing the reaction path. The experimental result was a CO yield of 398.87 μmol g^−1^ h^−1^ with a selectivity close to 100%.

**Figure 5:**
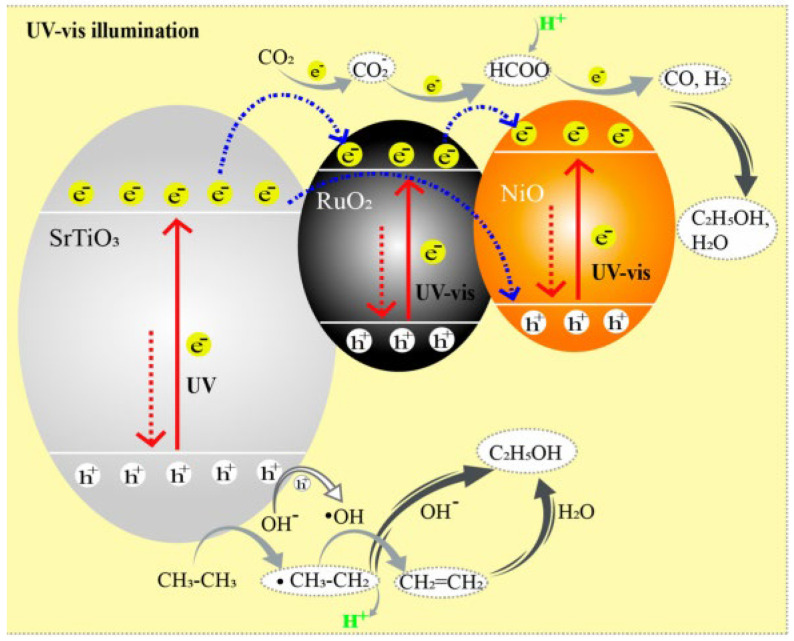
Reaction pathways and mechanisms for the preparation of ethanol, using CO_2_ and C_2_H_6_ as the reactants [73].

**Figure 6:**
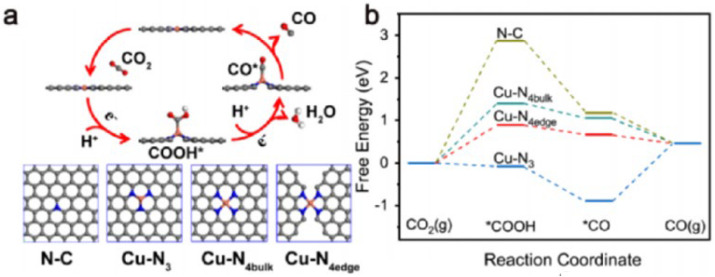
(**a**) The CO_2_ synthesis CO2RR reaction pathway and the optimized catalyst model. The gray, blue, and orange shapes represent the C, N, and Cu atoms, respectively. (**b**) Free energy diagrams for CO2RR [74]. Copyright 2021, Elsevier. * Active sites on the catalyst surface.

In general, such defect engineering could result in the formation of a differential charge distribution of polarized atom pairs. The polarized atom pairs have the potential to induce the adsorption of intermediates, such as methane and methanol, due to charge distribution differentiation, thereby enabling distinctive carbon-carbon bond formation processes, as reported in previous studies [73,74]. Figure 5 shows that the reaction pathways and mechanisms for the preparation of ethanol from CO_2_ and C_2_H_6_ [73]. Figure 6 depicts the CO_2_ synthesis CO2RR reaction pathway and the optimized catalyst model [74]. Therefore, a variety of catalysts need to be developed for the production of valuable products such as ethanol, ethane, or two-carbon hydrocarbons. Currently, some scholars have already converted CO_2_ to ethanol [75]. It is believed that in future research, new catalysts will be developed for the production of substances such as C3 and C4.

## 6. Conclusions

This paper aims to investigate the role and impact of defect modulation in the photocatalytic reduction of CO_2_. By analyzing the effects of different types of defects (e.g., metallic and non-metallic defects) on the performance of photocatalysts, the mechanisms of the influence of defect type, concentrations, and distribution on CO_2_ reduction efficiency, selectivity, and stability were revealed. In addition, the optimization methods of defect regulation strategies were discussed in this paper, with a view to providing theoretical guidance and experimental basis for the design and preparation of efficient and stable photocatalysts.

1. Employing advanced analytical techniques such as electron paramagnetic resonance (EPR), X-ray absorption near-edge structures (XANES), and extended X-ray absorption fine structures (EXAFS), the electronic structure and the intricate local coordination environment surrounding the defects can be meticulously delineated. This fine-level analysis facilitates careful study of the reaction process at the microscopic scale, revealing the complex ways in which defects affect the performance of photocatalysts. The subtle nuances of how defects can either enhance or impede photocatalytic activity can be uncovered by dissecting the reaction mechanisms at such a fundamental level. This profound understanding is crucial for the strategic manipulation of defects to optimize the efficiency of photocatalytic systems. It can achieve the customization of defect characteristics to achieve the desired photocatalytic performance, thereby driving innovation in the development of advanced photocatalytic materials.
2. With the aid of advanced computational methods and simulation software, the existence and distribution of defects in different photocatalysts can be simulated, and their impact on photocatalytic reaction activity can be predicted. This can not only reveal the influence of defects on the electronic structure and band structure of photocatalysts but also predict the impact of defects on the performance of photocatalysts, such as in the light absorption, separation, and transport of photogenerated carriers. Theoretical simulations allow for the identification of potential photocatalyst candidates, which can then be experimentally verified. By comparing the simulation results with experimental data, a more accurate understanding of how defects affect photocatalytic performance is obtained, thereby guiding the design and optimization of novel efficient photocatalysts. Simultaneously, based on the simulation and experimental findings, improvements in composition, structure, and preparation methods can be made to enhance the overall photocatalytic performance.
3. The application of the photocatalytic reduction of CO_2_ into high-value fuels is very promising, but unfavorable factors such as low spectral utilization and the short lifetime of photogenerated charges have been restricting the industrial application process. Therefore, in order to meet production requirements, it is necessary to develop a low-cost and simple synthesis method with a simple preparation process to prepare highly efficient and stable photocatalysts, which will make it possible to achieve practical applications and energy sustainability in the field of photocatalysis. The distribution of defects is important in the performance of photocatalysts. Uniformly distributed defects contribute to the separation efficiency of photogenerated carriers and the stability of the photocatalysts. For example, uniformly distributed defects could be formed on the surface of photocatalysts by specific synthesis methods, such as via solution or template methods. These methods help to improve the activity and selectivity of the photocatalyst while maintaining long-term stability.

## Figures and Tables

**Table 1:** Standard redox potentials for some specific reactions in the reduction of CO_2_ [36].

Specific Reaction	Redox Potentials *E*° (V) versus SHE at pH = 7
CO + *e^−^* → CO^−^	−1.9
CO_2_ + 2H^+^ + 2*e^−^* → HCOOH	−0.61
CO_2_ + 2H^+^ + 2*e^−^* → CO + H_2_O	−0.53
CO_2_ + 4H^+^ + 4*e^−^* → HCHO + H_2_O	-0.48
CO_2_ + 6H^+^ + 6*e^−^* → CH_3_OH + H_2_O	−0.38
CO_2_ + 8H^+^ + 8*e^−^* → CH_4_ + 2H_2_O	−0.24
2CO_2_ + 12H^+^ + 12*e^−^* → C_2_H_4_ + 4H_2_O	−0.34
2CO_2_ + 12H^+^ + 12*e^−^* → C_2_H_5_OH + 3H_2_O	−0.33
2CO_2_ + 14H^+^ + 14*e^−^* → C_2_H_6_ + 4H_2_O	−0.27
3CO_2_ + 18H^+^ + 18*e^−^*→ C_3_H_7_OH + 5H_2_O	−0.32
3CO_2_ + 20H^+^ + 20*e^−^* → C_3_H_8_ + 6H_2_O	−0.33
2H^+^ + 2*e^−^* → H_2_	−0.41
2H_2_O + 4*h^+^* → 4H^+^ + O_2_	0.82

**Table 2:** The role of defects in CO_2_ conversion and product selectivity.

Type of Defect	Defective Photocatalyst	Product	Yield and Selectivity	Roles of Defects on Photocatalysis	Mechanism	Ref.
Cation vacancy	ZnS	HCOOH	Selectivity up to 86.6%	Lowering the barrier of CO2RR and suppressing proton adsorption	Changing the electronic states of density	[41]
Cation vacancy	ZnIn_2_S_4_	CO	33.2 μmol g^−1^ h^−1^	Increasing the light absorption, improving the CO_2_ adsorption capacity, and enhancing surface hydrophilicity	Increased charge density	[42]
Metal vacancy	Ag-TiO_2_	CH_4_	16.0 ppm/g h	Promoting the separation of photo-induced electron-hole pairs	Forming a Schottky barrier and surface plasmon resonance	[43]
Metal vacancy	PtCu/TiO_2_	CH_4_	Selectivity up to 100%	Enhancing the adsorption/activation of CO_2_/CO and the further hydrogenation of CO	Reducing the activation energy barriers of *CO_2_ and *CHO and inhibiting the desorption of *CO	[44]
Metal vacancy	Co-CN	CO	94.9 umol/g/h	Reducing the energy barrier of CO_2_ adsorption/activation and promoting the	Strong interaction between electrons	[45]
Carbon vacancy	GCN	CO	4.18 mmol g^−1^ h^−1^	Enhancing CO_2_ adsorption/activation, upshifting the conduction band and elevating the charge carrier concentration and lifetime	Attenuating the exciton effect and facilitating charge carrier generation	[55]
Doping	Cu-TiO_2_	CH_4_	8.04 μmol g^−1^ h^−1^	Increasing visible-light absorption in the materials, and suppressing photogenerated electron-hole recombination	Serving as electron traps	[56]
Doping	O-doped g-C_3_N_4_	CH_3_OH	0.88 µmol g^-1^ h^-1^	Improving light utilization efficiency and CO_2_ affinity, and separation efficiency of photogenerated charge carriers	Optimizing the band structure	[60]

* Active sites on the catalyst surface.

## Data Availability

Data are contained within the article.
